# Cardiac Tamponade by Tack Fixation of a Hiatal Mesh. Should Tacks Still Be Used in the Diaphragm?

**DOI:** 10.7759/cureus.8416

**Published:** 2020-06-02

**Authors:** Ramon Vidrio Duarte, Eduardo Vidrio Duarte, Juan Gutierrez Ochoa, Luis H Ortega León, Carolina Solis Rojas

**Affiliations:** 1 Surgery, Hospital General de Mexico, Mexico City, MEX; 2 General Surgery, Hospital Angeles Metropolitano, Mexico City, MEX; 3 General Surgery, Hospital General de México "Dr. Eduardo Liceaga", Mexico City, MEX; 4 General Medicine, Universidad Anahuac Norte, Mexico City, MEX

**Keywords:** cardiac tamponade, surgical mesh, hiatal hernia, surgical fixation devices

## Abstract

Since the first successful use of mesh in hernia surgery, the development and progress in materials, techniques, and procedures have increased exponentially; consequently, surgeons started to use meshes for hiatal hernia repair to prevent postoperative hernia recurrences and complications. Nonetheless, there are alarming reports in literature concerning cardiac tamponade as an apparently rare complication of hiatal mesh placement, especially when fixation is performed with tacks.

A 50-year-old female diagnosed with gastroesophageal reflux disease undergoes an elective laparoscopic Nissen fundoplication and hiatal hernia repair with tack fixation of the mesh; on the fourth postoperative day she was readmitted with cardiac tamponade diagnosed via echocardiography, and CT scan showed proximity of the tacks to the pericardium. She underwent a failed attempt of ultrasound guided pericardiocentesis (PC), therefore, a pericardial window was performed.

The ideal method for diaphragmatic mesh fixation is still controversial. Some recent articles alert of this potential risk; although the manufacturers contraindicate the use of tacks in the diaphragm, one-third of surgeons prefer this method.

## Introduction

Since the first successful use of mesh in hernia surgery, the development and progress in materials, techniques, and procedures have increased exponentially. Nowadays, tension-free repairs are worldwide implemented as the first choice option for hernia repair surgery; consequently, surgeons started to use meshes for hiatal hernia repair, as it has proven to be a safe and effective procedure, including the laparoscopic antireflux surgery preventing postoperative hernia recurrences, especially on big hiatal defects, as well as lower complication rates (2%) [1-2]. Nonetheless, there are alarming reports in literature concerning cardiac tamponade as an apparently rare complication of hiatal mesh placement, especially when fixation is performed with tacks.

## Case presentation

A 50-year-old female with gastroesophageal reflux disease treated with omeprazole, with an endoscopy results of type I hiatal hernia, suggestive findings of Barrett's esophagus, chronic superficial gastritis and polyps on gastric corpus and fundus, along with a manometry revealing a nonspecific esophageal disorder, underwent an elective laparoscopic Nissen fundoplication with polytetrafluoroethylene mesh repair of the esophageus hiatus with no intraoperative complications. The patient was discharged on the first postoperative day and coursed asymptomatic for three days, reporting only mild epigastric pain and adequate food intake.

On the fourth postoperative day, she went to the ER after a witnessed syncopal episode with hypotension of 70/40 mmHg, tachycardia of 147 bpm, tachypnea of 30 bpm, and 35.5ºC of temperature. She was pale, notably, her external jugular veins were distended, with muffled heart sounds, normal abdominal examination showed no alteration on surgical wounds and she had delayed capillary refill; she integrated Beck’s triad, thus, cardiac tamponade was suspected, fluid therapy with lactate ringer’s solution was provided with adequate hemodynamic response, also omeprazole, analgesics, and antibiotics were administered.

**Figure 1:**
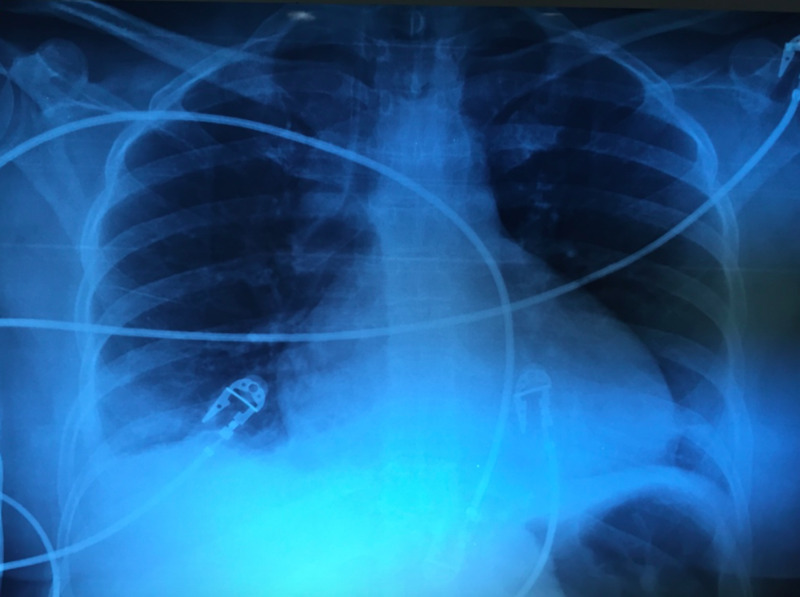
Chest X-ray. Pericardial effusion, water bottle sign.

**Figure 2:**
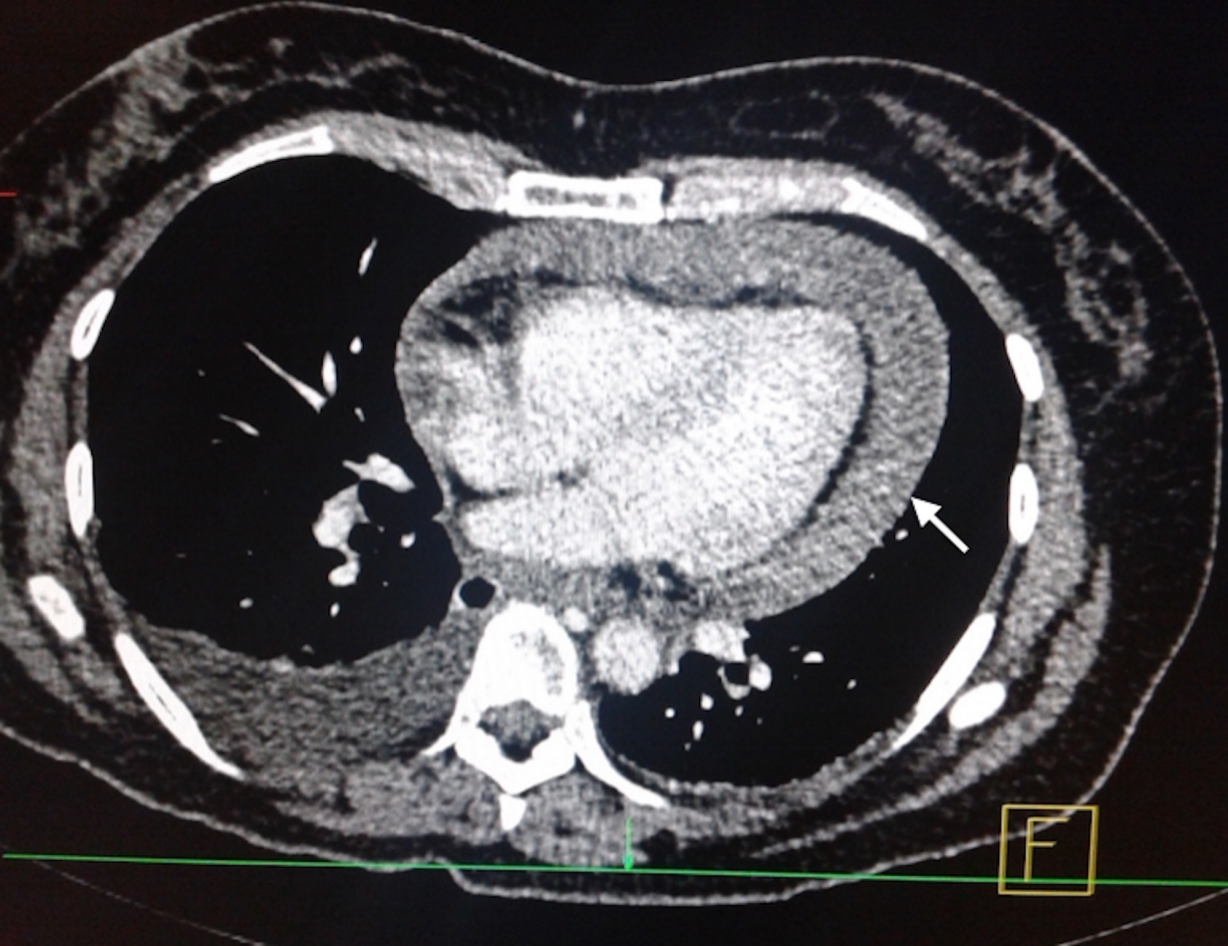
CT scan, axial plane. Pericardial effusion estimated in 400 cc (white arrow).

**Figure 3:**
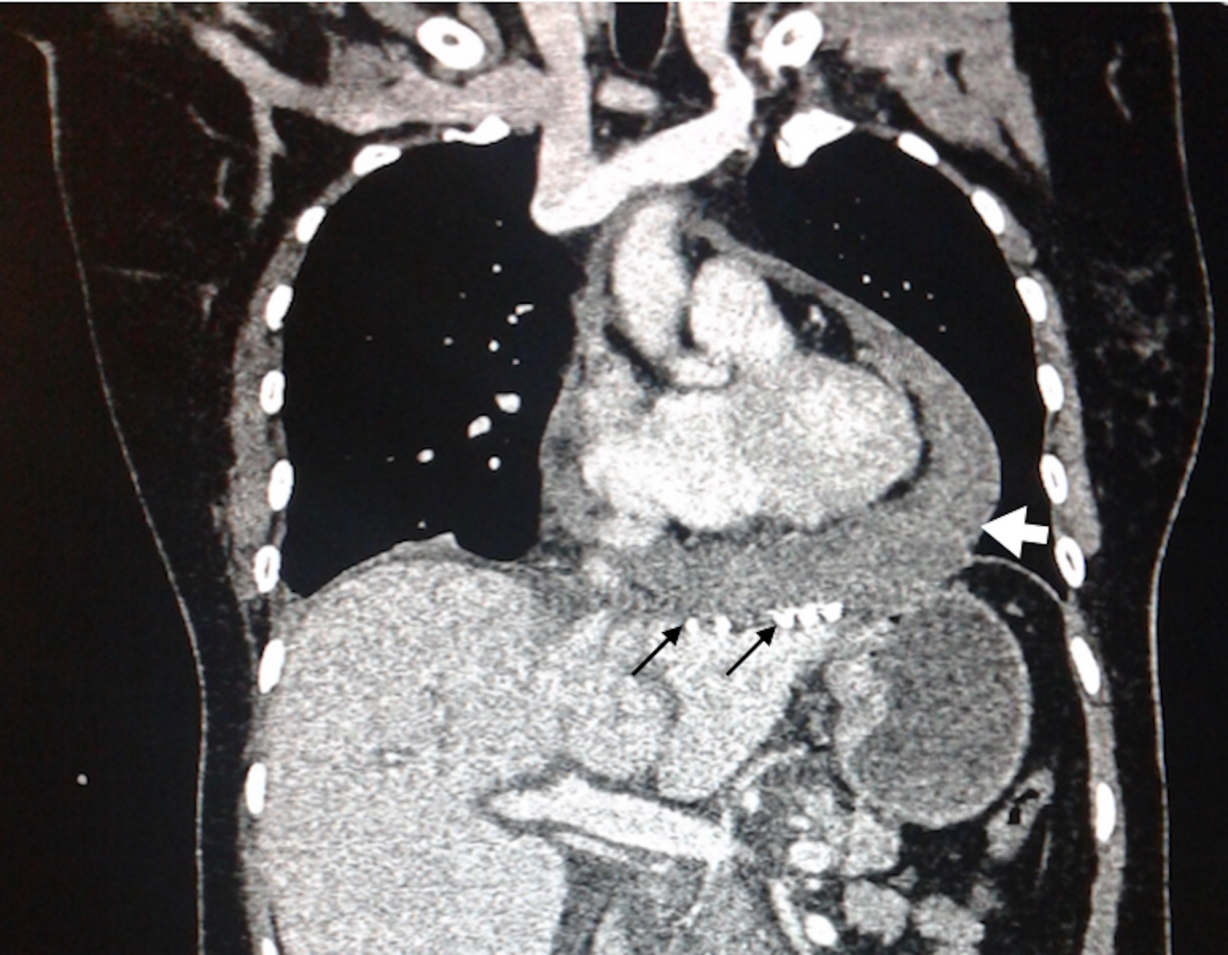
CT scan, coronal plane. Close approximation of tacks to the pericardium (thin black arrows) causing pericardial effusion (thick white arrow).

Her laboratory tests were: hemoglobin 11.5 g/dL, hematocrit 33.8%, platelet count of 370,000/HPF, leucocytes 12.8/HPF, neutrophils 8.9/HPF, D-dimer 1094 ug/L, creatinine 0.98 mg/dL, glucose 305 mg/dL. Electrocardiogram (ECG) showed sinus tachycardia and low voltage QRS complexes, the chest X-ray revealed pericardial effusion with a characteristic “water bottle sign” (Figure 1), echocardiography reported 400 cc pericardial effusion coupled with right ventricular diastolic dysfunction and a left ventricle intramural hematoma. The CT scan demonstrated a close proximity of the tacks, used on her prior surgery for hiatal mesh fixation, to the inferior surface of the hearth (Figure 2), with pleural and pericardial effusion (Figure 3).

Once initial stabilization was achieved, the patient went to the ICU and underwent a failed attempt of ultrasound guided pericardiocentesis (PC), attributable to the hiatal mesh presence; therefore, the patient went into surgery, where a pericardial window was performed via thoracotomy; 300 cc of hematic pericardial effusion were drained along with approximately 600 cc of pleural effusion, two pleural drains were left in place.

The patient’s postoperative course was completely uneventful; an echocardiogram was performed 24 hours after surgery showing no pericardial effusion, thus the drains were removed when a minimal output was achieved on postoperative days 4 and 7, she was discharged asymptomatic 13 days after her intervention.

## Discussion

Cardiac tamponade is characterized by an accumulation of fluid in the pericardium, resulting in compression of all cardiac chambers and therefore compromising the entire circulatory blood flow [3]. This disease as a complication of hiatal surgery is a relatively rare entity but with a significant mortality rate; there are few reports on literature regarding this surgical complication, approximately 30 cases with the first of them just being made 10 years ago [4], nevertheless it may be overlooked and under-reported, mainly because mild initial symptoms overlap in context of a recent surgical procedure. Moreover, in most of the reported cases, this pathology is suspected only when low cardiac output symptoms present, as it happened in our case, where the patient integrated Beck’s triad at her admission; hence some authors have expressed their concern, predominantly on case reports and some recent literature reviews.

On a recent review by Çalıkoğlu et al. the authors found a 33.3% mortality rate for iatrogenic cardiac tamponade (ICT) [5]; this correlates with previously reported rates varying from 37.5% to 66%, however, there may be higher incidence and mortality rates, as it may be under-reported due to legal implications and lack of diagnosis. In this study, they also reported that mesh placement was responsible for nine out of 10 cases, most of them fixed with tacks; in addition, Köckerling et al. revealed that 22 out of 25 reports were secondary to tack fixation [6].

By understanding the anatomy of the diaphragm, a possible explanation for this pathology could be inferred; the thickness of the diaphragm varies from 1.5 to 5.4 mm, however, during surgical procedures, the patient positioning associated with the pneumoperitoneum, causes the diaphragm to be tighter, and therefore, in more proximity to the pericardium, specially at the central tendon where the thickness is between 2.9 and 3.0 mm [7].

The average tack device ranges from 3.8 to 7.0 mm, varying amongst manufacturers. In all of these product´s specifications, they emphasize that a major contraindication for the use of their device is a close vicinity to major vascular structures, including the diaphragm. Moreover, the SAGES guidelines for laparoscopic ventral hernia repair state that tack use should be avoided over the umbilicus; meanwhile, the SAGES guidelines for hiatal hernia mention that insufficient evidence exists for a recommendation regarding fixations techniques. Nevertheless, they should be used with caution, particularly tacks in order to prevent aortic or pericardial injuries [8], as inspite of all these warnings, tack fixation is the second preferred technique of mesh anchorage amongst surgeons; on a SAGES survey of 261 surgeons, the most common fixation technique was suture anchorage (56%), followed by tack fixation (23.9%) and remarkably, fibrin glue, as the safest method is the most unusual technique (1.1%) [9].

Echocardiography and tomography are efficient diagnostic tools in ICT, as the clinical manifestations might be mistaken with other complications or even with normal recovery after surgery, the use of these diagnostic tests should not be delayed [10]. Regarding treatment, PC is the immediate treatment which can be performed in the ER, although Çalıkoğlu et al. reported that open sternotomy was needed in 18 of the 23 patients as a first approach or either as a second procedure [5].

## Conclusions

Although cardiac tamponade as a complication of hiatal mesh fixation, especially with tacks, is an infrequent entity, care should be taken in order to avoid it, due to its high mortality rate and the fact that it is a preventable complication. There is plenty of information with regard to avoiding the use of tacks placement on the diaphragm, which appears as a contraindication in all of these device's specifications, however many surgeons continue to use them as their preferred fixation method.
